# Preparation of a whole cell catalyst overexpressing acetohydroxyacid synthase of *Thermotoga maritima* and its application in the syntheses of α-hydroxyketones

**DOI:** 10.1038/s41598-020-72416-6

**Published:** 2020-09-21

**Authors:** Yan-Fei Liang, Le-Tian Yan, Qiao Yue, Ji-Kui Zhao, Cai-Yun Luo, Feng Gao, Heng Li, Wen-Yun Gao

**Affiliations:** College of Life Sciences, Northwest University, 229 North Taibai Road, Xi’an, 710069 Shaanxi People’s Republic of China

**Keywords:** Biocatalysis, Enzymes, Biocatalysis

## Abstract

The large catalytic subunit of acetohydroxyacid synthase (AHAS, EC 2.2.1.6) of *Thermotoga maritima* (TmcAHAS) was prepared in this study. It possesses high specific activity and excellent stability. The protein and a whole cell catalyst overexpressing the protein were applied to the preparation of α-hydroxyketones including acetoin (AC), 3-hydroxy-2-pentanone (HP), and (*R*)-phenylacetylcarbinol (*R-*PAC). The results show that AC and HP could be produced in high yields (84% and 62%, respectively), while *R*-PAC could be synthesized in a high yield (about 78%) with an *R*/*S* ratio of 9:1. Therefore, TmcAHAS and the whole cell catalyst overexpressing the protein could be practically useful bio-catalysts in the preparation of α-hydroxyketones including AC, HP, and *R*-PAC. To the best of our knowledge, this is the first time that bacterial AHAS was used as a catalyst to prepare HP with a good yield, and also the first time that TmcAHAS was employed to synthesize AC and *R*-PAC.

## Introduction

Acetoin (3-hydroxy-2-butanone, AC), a representative of α-hydroxyketone is always a versatile synthon in both synthetic organic chemistry and medicinal chemistry. Moreover, AC can also be used as a flavor enhancer of butter, cheese, coffee and nut containing food because of its pleasant odor. Up to now, quite many biotransformation methods have been established to produce AC, mainly through oxidation of 2,3-butanediol by 2,3-butanediol dehydrogenase of different microorganisms or reduction of diacetyl by carbonyl reductase^1–4^. A cell-free cascade for preparation of AC using thermostable acetolactate synthase (ALS) from *Caldicellulosiruptor owensensis* and α-acetolactate decarboxylase from *Bacillus subtilis* has also been reported^5^.

**Figure 1:**
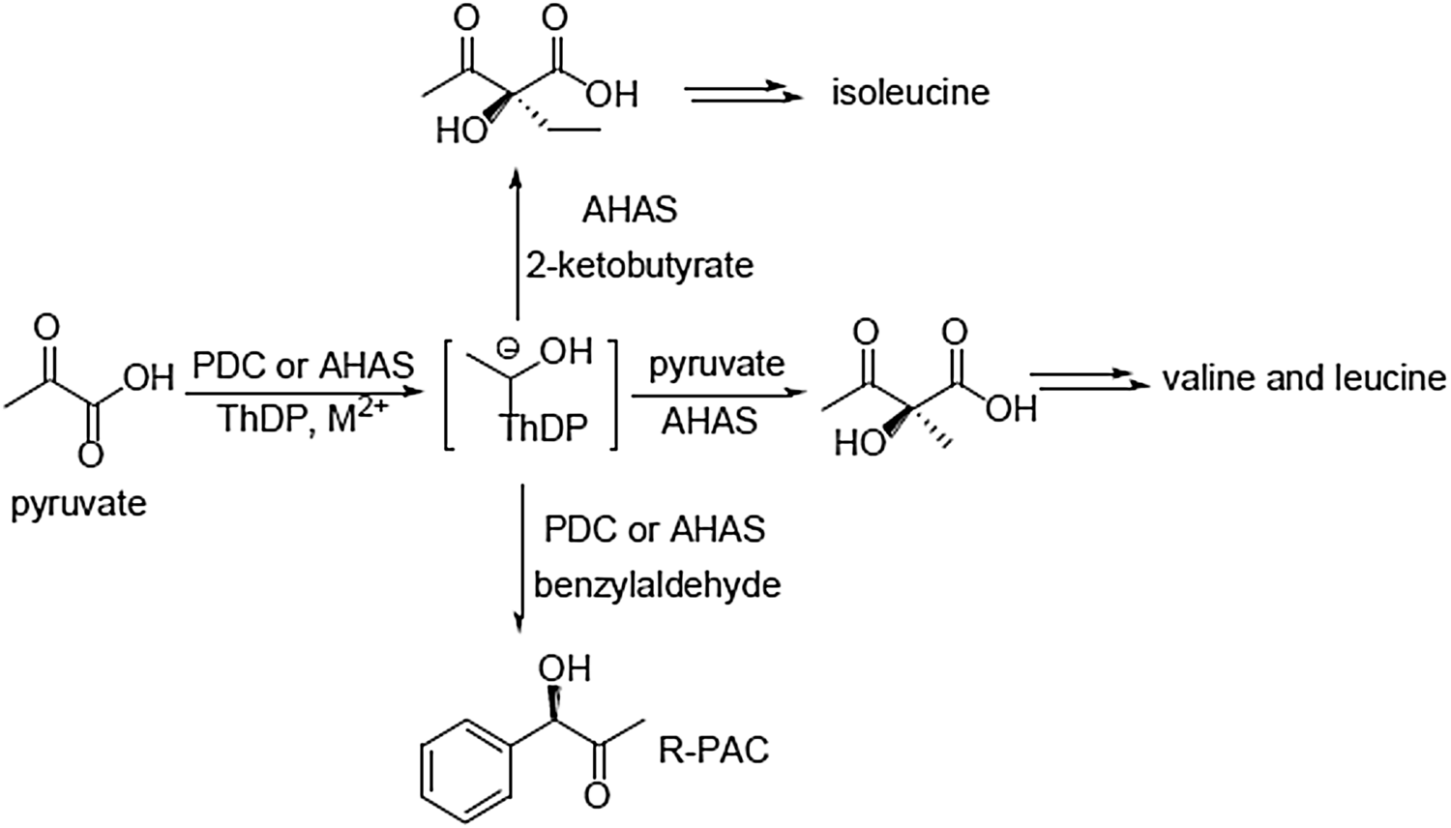
Enantiospecific condensation reactions catalyzed by AHAS.

*R*-Phenylacetyl carbinol (*R*-PAC) is another important α-hydroxyketone used as a chiral building block to prepare many drugs showing α- and β*-*adrenergic traits^6^. *R*-PAC has been produced generally through fermentation in which pyruvate condenses with benzaldehyde (BA) (Fig. 1)^7^. To improve the method, Meyer et al.^8^ mutated pyruvate decarboxylase (PDC) of *Zymomonas mobilis* and found one mutant was able to convert pyruvate and BA to *R*-PAC with very good results (yield ~ 98%, ee 98.4%). But the low catalytic efficiency of the mutant largely prevents its practical application. Recent studies have shown that PDCs have some advantages in the formation of intramolecular C–C bonds from substituted benzaldehydes or aliphatic dialdehydes^9,10^.

Acetohydroxyacid synthase (AHAS, EC 2.2.1.6) is one of the key enzymes involved in the biosynthesis of branched-chain amino acids including valine, leucine, and isoleucine. It catalyzes the reaction of two molecules of pyruvate or pyruvate with 2-ketobutyrate to generate (*S*)-2-acetolactate or/and (*S*)-2-aceto-2-hydroxybutyrate, respectively (Fig. 1)^11^. Furthermore, AHAS also possesses the ability to catalyze the condensation between pyruvate and BA to form *R*-PAC or BA derivatives to produce PAC analogues^12–19^.

Up to now, three different AHAS isozymes (I, II, and III) have been characterized in *Escherichia coli*^20^. The bacterial AHASs normally consist of two catalytic subunits (CSU) and two regulatory subunits (RSU)^11^. Although the CSU of *E. coli* AHAS I is active, its activity is low and is unstable alone^21–23^. In one of our previous researches, we prepared glutathione S-transferase-tagged CSU of *E. coli* AHAS I which is stable and active and used it to stereoselectively synthesize *R*-PAC with a yield of 80.6% and an *ee* value of higher than 98%^14^. The drawback of the procedure is that the preparation of the protein is always in low yield.

Since its identification in the middle of 1980s, *Thermotoga maritima* has become an important model hyperthermophile and its enzymes are attractive for biotechnology applications because of their extreme thermostability. A number of potential uses of carbohydrate active enzymes from the microorganism have been described^24^. In one of our ongoing projects, we need thermostable enzymes to explore their applications in organic synthesis, especially in the stereopecific C–C bond formation of the acyloin-like reaction. We thus selected the AHAS of *T. maritima* to carry out our research. Although this protein has been overexpressed^25^, but its application in organic synthesis has not been characterized. Herein we would disclose all the experimental details.

## Results and discussion

### Expression and purification of recombinant TmcAHAS

**Figure 2:**
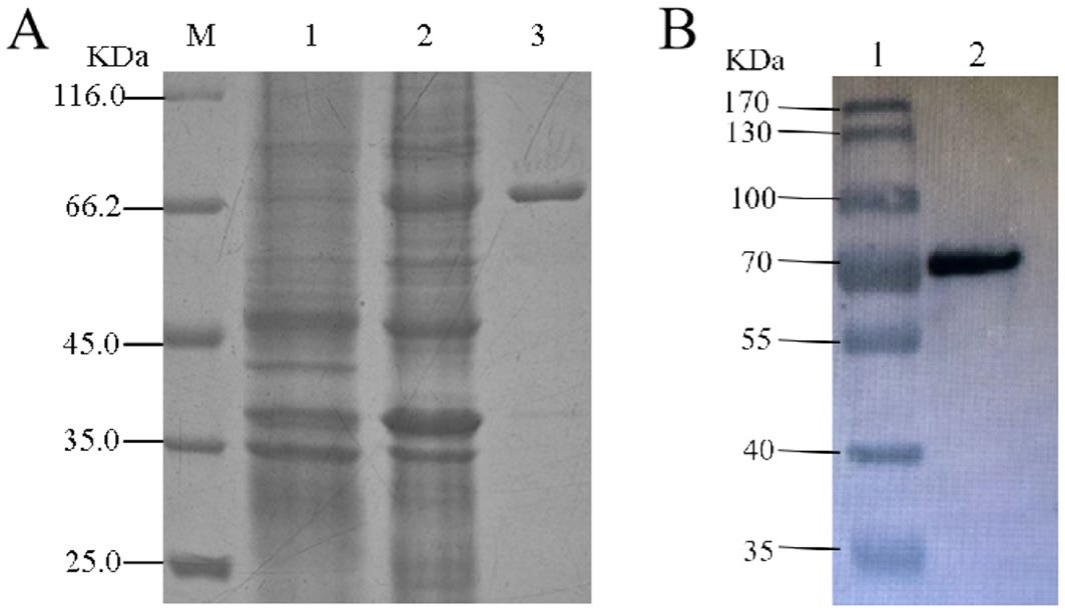
Purification of TmcAHAS and western-blotting confirmation. (**A**) SDS-PAGE results, lane M: unstained protein molecular weight marker; lane1: Cell extract of *E. coli* Rosetta(DE3) harboring pET28a-TmcAHAS before induction with IPTG; lane 2: after induction with IPTG; lane 3: purified His_6_-TmcAHAS. (**B**) Western-blotting results, lane 1: prestained protein molecular weight marker; lane 2: Ni^2+^-His affinity columns purified His_6_-TmcAHAS.

The result of the construction of TmcAHAS expression vector was depicted in the Figure S1 of the Supporting Information (SI). The recombinant plasmid pET28a-TmcAHAS was transformed and expressed in *E. coli* Rosetta(DE3) as described above. The enzyme was expressed in the soluble fraction at a significant level when it was induced at 25 °C with 0.5 mM IPTG (Fig. 2A, lane 2). TmcAHAS with His_6_-tag was purified from the soluble fractions of cell extract by Ni–NTA affinity chromatography. The active fractions eluted with 250 mM imidazole were collected and determined by 12% SDS-PAGE, revealing that one band with a molecular weight of about 68 kDa is almost same as predicted (Fig. 2A, lane 3). The overall yield of purified TmcAHAS was about 14 mg per liter of culture (ca 4 g cell pellet). So it could be calculated that the content of TmcAHAS in the whole cell catalyst was 3.5 mg/g cell (wet weight). The expression of the recombinant TmcAHAS was further confirmed by His_6_-immunoblot (Fig. 2B).

### Optimum conditions for TmcAHAS assay

In the UV determination of AHAS activity, a decarboxylation step executed by an acid or acetolactate decarboxylase to convert acetolactate to AC is normally mandatory^24^. We found that in TmcAHAS assay which was carried out at 80 °C, this step could be omitted because acetolactate formed in the assay could be completely thermo-decarboxylated to AC. Therefore no acid or decarboxylase decarboxylation process was included in the activity assay of TmcAHAS. We observed that the protein exhibited its best activity in 50 mM phosphate buffer at a pH value of 8.0, an assay temperature of 80 °C, and under such conditions highest conversion of pyruvate was obtained after an assay time of 1 h. The optimized conditions for the enzyme are basically identical with the published data^25^. The experimental details were given in Figure S2 in the SI.

### Stability of TmcAHAS

**Figure 3:**
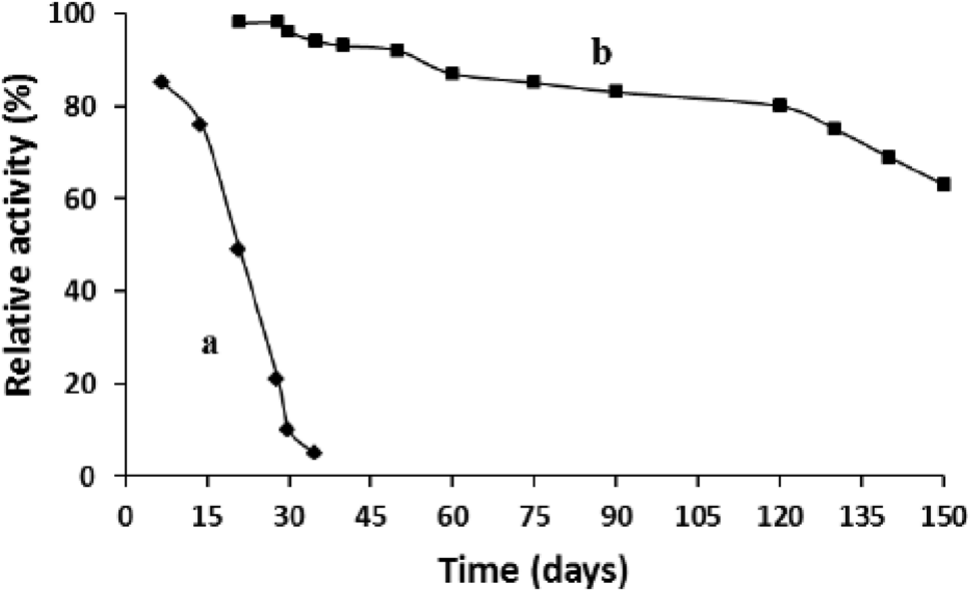
Stability of TmcAHAS stored in different buffers at − 80 °C. Line a: in 50 mM Tris–HCl buffer (pH 8.0, containing 5 mM DTT and 20% glycerol); Line b: in 50 mM phosphate buffer (pH 8.0, containing 5 mM DTT and 20% glycerol).

Because it has been reported that although the CSU of *E. coli* AHAS possesses full catalytic machinery, both of its stability and turnover numbers were sharply decreased in the absence of the RSU^21–23^, the stability issue of TmcAHAS prepared in this study became into our first concern after its activity had been confirmed and optimized. The purified protein was kept at − 80 °C in two different buffers, respectively and its activity were measured at different time points. The determination displayed that the enzyme stored in the Tris–HCl buffer (Fig. 3, line a) could maintain about 80% of its activity after two weeks, then the activity dropped quickly and after five weeks, the enzyme almost lost all of its ability to catalyze the reaction. Fortunately, the protein stored in the phosphate buffer gave an encouraging result (Fig. 3, line b). During the first 4 months, its activity decreased very slowly and after maintained at − 80 °C for 4 months, more than 80% of its activity remained. Even after 5 months, around 60% of its total activity was still available. These results showed that TmcAHAS is much more stable than CSU of *E. coli* AHAS, it can be kept at − 80 °C in phosphate buffer for at least 4 months without significant loss of its activity. In addition, these results also indicated that phosphate buffer is a better choice for the storage of TmcAHAS, the reason for which deserves further investigation.

### The kinetic parameters of TmcAHAS

The kinetic parameters of TmcAHAS were determined using the photometric method and the results were listed in Table 1 (also Figure S3 in SI). We can find that while the Michaelis constant of TmcAHAS for pyruvate is in the same level as those of the CSUs of the other bacterial AHASs, the specific activity of TmcAHAS determined in this study is a bit bigger than that of the CSU of the *E. coli* AHAS I and is 5 to 110-fold larger than that of the CSUs of the other bacterial AHASs. These kinetic traits plus its stability displayed that TmcAHAS possesses a high potential in practical utilization.

**Table 1:** The kinetic parameters of TmcAHAS and the CSUs of microbial AHASs.

Sources of AHAS	Specific activities (μmol min^−1^ mg^−1^)	Km for pyruvate (mM)	References
*E. coli* (AHAS I)	6–12 (63.6)^a^	(4.8)	^22^
*E. coli* (AHAS II)	Trace (20)	(5.0)	^21,26^
*E. coli* (AHAS III)	0.23 (2.7)	86 (11.5)	^21^
*M. tuberculosis*	2.8 (4.6)	2.76 (1.56)	^27^
*E. coli* K12 (AHAS I)	1.5	4.15	^28^
S*. sonnei*	0.12	8.01	^29^
*H. influenza*	1.5	9.2	^30^
*S. cerevisiae*	6.8 (49)	8.6 (18.1)	^31^
*T. maritima*	134	16.4	^25^
*T. maritima*	13.4 ± 1.1	19.1 ± 1.6	This study

^a^Data in the parentheses represent the corresponding parameters of holoenzyme.

### Preparation of AC and 3-hydroxy-2-pentanone (HP)

Based on the above characterization, we started to evaluate the utility of TmcAHAS in the construction of new C–C bond, namely the preparation of certain α-hydroxyketones. Generally, a decarboxylation step that is executed by acetolactate decarboxylase is necessary in the synthesis of AC using pyruvate as a substrate^5^. But as mentioned above, acetolactate generated in TmcAHAS catalyzed reaction could be simultaneously decarboxylated to AC. Therefore no decarboxylase was needed in the process. The reaction was performed under the optimum conditions and monitored by GC. After 50 min, the yield of AC measured by GC reached about 92% and the datum remained almost unchanged after 10 more minutes. Thus the reaction was terminated and the mixture was cooled to rt on ice. After removal of the precipitate through centrifugation, ethyl acetate extraction, and silica gel column isolation, AC was obtained as colorless oil with satisfactory yield (18.5 mg, isolated yield 84.1%). Further chiral GC analysis showed that the *R*/*S* ratio of the product was about 1 (Fig. S4 in the SI), indicating that racemic AC was produced in the reaction.

The preparation of HP was carried out under the same conditions as for AC other than that one more substrate, 2-ketobutyrate was added. To maximize the production of HP, three pyruvate to 2-ketobutyrate ratios (100 mM to 50 mM, 50 mM to 50 mM, and 50 mM to 100 mM) were determined by GC and the results (Fig. S5 in the SI) showed that at a ratio of 100 mM to 50 mM, AC was the major product with a yield of about 52% and HP was almost equally produced. At a reversed ratio, the production of both compounds reduced, which could be resulted from the inhibition of 2-ketobutyrate at a high concentration. A 50 mM to 50 mM ratio led to a reasonable result with HP yield being more than 65% and AC yield less than 10%. Therefore this ratio was selected in the preparation of HP. Then the reaction was carried out under the optimum conditions and monitored by GC. The GC determination displayed that the reaction reached its completion after 1 h incubation. Following the identical workup step, HP was acquired as a colorless oil with a good yield (> 60%). The product HP was assayed by chiral GC and the result showed (Fig. S6 in the SI) that HP produced in the reaction was racemic as well (*R*/*S* = 1).

3-Hydroxy-2-pentanone (HP). Colorless oil (15.8 mg, isolated yield 62.0%). EI-MS (70 eV) *m/z* (%) 102 (2) [M]^+^, 84 (7) [M-H_2_O]^+^, 73 (7), 59 (100), 43 (53), 41 (22). ^1^H-NMR (400 MHz, CDCl_3_) δ 4.23 (dd, J = 7.6, 5.1, 1H, CH), 2.19 (s, 3H, Me), 1.97 (m, 1H, CH_2_), 1.79 (m, 1H, CH_2_), 1.02 (t, J = 7.1, 3H, Me). ^13^C-NMR (100 MHz, CDCl_3_) δ 206.32 (C=O), 78.09 (CH), 26.71 (CH_2_), 24.55 (CH_3_), 10.71 (CH_3_).

The whole cell catalyst overexpressing TmcAHAS was also employed to mediate the reactions preparing AC and HP and almost same results (both in yield and enantioselectivity) were acquired. Therefore use of the whole cell catalyst was preferred in the preparation of AC and HP.

HP is a volatile natural product isolated from *Corynebacterium glutamicum* that is related to the biosynthesis of pyrazines^32^. Just like AC and PAC, HP may also be a useful α-hydroxy carbonyl that can be used as a synthon for the synthesis of natural products such as antitumor agents, antibiotics, pheromones, and sugars^33,34^. But the chemical synthesis of the compound was in extremely low yields (< 10%)^32,34,35^. As described in the Introduction, AHAS can catalyze the reaction of pyruvate to form (*S*)-2-acetolactate and this reaction has been widely applied to the preparation of AC. It can also mediate the reaction between pyruvate and 2-ketobutyrate to produce (*S*)-2-aceto-2-hydroxybutyrate that is the precursor of HP. But the alternative reaction has never been extended to the synthesis of this α-hydroxylated 2-pentanone. The possible reasons could be (1) although AHAS I of *E. coli* possesses excellent activity (Table 1), it shows no acceptor substrate preference (Specificity ratio R = 1)^36^. This means that if AHAS I is used as a catalyst to prepare HP, AC can be produced as a byproduct in almost same amount, which largely reduces the efficiency of the synthesis; (2) the other enzymes like AHASs II and III of *E. coli* truly exhibit high acceptor substrate preference (Specificity ratio R = 180 and 60, respectively)^36^, but their catalytic activities (Table 1) are too low to be employed as practical catalysts in the organic synthesis. Thus we tried in this study to set up an enzymatic method to prepare HP using TmcAHAS as a catalyst. The results described above definitely show that TmcAHAS holds not only high activity, but also good 2-ketobutyrate preference and can be used to prepare α-hydroxy carbonyls such as AC and HP with high efficacy. The drawback is that the syntheses showed no stereoselectivity, which might be resulted from the thermo-decarboxylation step.

### Synthesis of R-PAC

It is a very important function of *E. coli* AHAS to catalyze the enantiospecific condensation of pyruvate and BA to form *R*-PAC^10^. We therefore checked whether TmcAHAS also possesses this activity. Firstly, the reaction conditions including reaction time, the ratio of substrates, the maximum concentration of BA, and the content of DMSO were optimized. The data (Fig. S7, SI) showed that maximum production of *R*-PAC could be obtained when 50 mM pyruvate, 40 mM BA, and 5% DMSO were used and the reaction was incubated for 1 h. Secondly, the efficiency of purified TmcAHAS and the whole cell catalyst was evaluated as well. The results indicated that when same amount of protein was utilized, the whole cell catalyst always produced more product than the pure enzyme and the assay using 14.3 mg whole cell catalyst in a total volume of 0.5 mL (containing about 50 μg TmcAHAS) generated the most *R*-PAC which was about 20% higher than the assay using 50 μg TmcAHAS (Fig. S8, SI). This is distinct from what we observed in the synthesis of AC and HP in which the use of pure enzyme and the whole cell catalyst made no difference. A reasonable explanation is yet obscure. Anyway, the use of the whole cell catalyst is a must in the preparation of *R*-PAC. Adopting the optimized conditions established in above experiments, *R*-PAC was prepared in an excellent isolated yield (> 80%). Furthermore the stereoselectivity of the reaction was assessed through chiral GC measurement and the result showed that an *R*/*S* ratio was about 3:1, indicating a very low enantiospecificity of the reaction. To improve the enantioselectivity of the reaction, we tried to carry out the reaction at a lower temperature (50 °C), a longer time (overnight), and more catalyst (200 mg), the product could be produced with an isolated yield of about 78% and an *R*/*S* ratio of about 9:1 (Fig. S9, SI). These data showed obviously the whole cell catalyst overexpressing TmcAHAS could be a practically applicable bio-catalyst in the preparation of *R*-PAC in high yield.

*R*-Phenylacetyl carbinol (*R*-PAC). White powder (24.7 mg, yield 82.3%). HRESI-MS (negative mode) *m/z*: 149.0605 (100%) [M-H]^−^, cacl. 149.0603; ^1^H-NMR (600 MHz, CDCl_3_): δ 7.35–7.41 (m, 5H, aromatic), 5.10 (s, 1H, CH), 4.23 (brs, 1H, OH), 2.05 (s, 3H, Me). ^13^C-NMR (100 MHz, CDCl_3_): δ 207.21 (C=O), 137.98 (aromatic CH), 129.16 (aromatic CH × 2), 128.76 (aromatic CH), 127.46 (aromatic CH × 2), 80.22 (CH), 25.39 (CH_3_). *R*/*S* ratio: 3:1 (80 °C, 1 h); 9:1 (50 °C, overnight).

In summary, we prepared and characterized in this study the TmcAHAS and also a whole cell catalyst overexpressing the enzyme. The evaluation of the protein revealed that it possesses a good specific activity and excellent stability. Using the pure enzyme and the whole cell catalyst as tools, we prepared α-hydroxyketones including acetoin, 3-hydroxy-2-pentanone, and (*R*)-phenylacetylcarbinol with satisfactory results. Therefore we would say that TmcAHAS and the whole cell catalyst could be practically applicable bio-catalysts in the preparation of α-hydroxyketone compounds.

## Materials and methods

### Materials

The genomic DNA of *T. maritima* MSB8 was purchased from Deutsche Sammlung von Mikroorganismen und Zellkulturen GmbH (Braunschweig, Germany). *E. coli* Rosetta(DE3), prokaryotic expression vector pET28a were purchased from Novagen (Madison, WI, USA). Restriction endonucleases were purchased from New England Biolabs. T4 DNA ligase, Taq DNA polymerase, Gel DNA extraction kit, Plasmid DNA preparation kit, and other molecular biology reagents were obtained from Takara Biotech. Co. (Dalian, China). Bradford protein assay kit was purchased from Bio-Rad Laboratories (Hercules, USA). Anti His-tag rabbit polyclonal antibody and alkaline phosphatase conjugated goat anti-rabbit IgG were from Bio Basic Inc. (Amherst, New York). Racemic AC was purchased from Aldrich (Sigma-Aldrich Shanghai, China). (*R*)-3-Hydroxy-2-pentanone (HP) was a kind gift from Prof. Dr. Viviana Heguaburu in Departamento de Química del Litoral, Universidad de la República, Uruguay. All chemicals and antibiotics were purchased from Sigma-Aldrich (USA) unless stated otherwise.

### Construction of the CSU of *T. maritima* AHAS (TmcAHAS) expression vector

The gene encoding TmcAHAS was PCR-amplified from genomic DNA with the following primers: GGAATTCCATATGATGCTTCTGGACGAGATC and CGCGGATCCTCATACTTTCCCCTCCCTAC (synthesized by Shanghai Sangon Biological Engineering Company). The underlined nucleotide sequences represent *Nde*I and *Bam*HI restriction endonuclease sites, respectively. The PCR reaction was performed in 50 µL mixture containing 5 µL of 10 × PCR buffer, 1.5 mM MgCl_2_, 200 µM of dNTP, 0.5 µM of each primers, 500 ng of *T. maritime* genomic DNA and 2.5 units of Taq DNA polymerase. Amplification was started with an initial activation step at 94 °C for 10 min, followed by 30 cycles, each consisting of 94 °C for 1 min, 65 °C for 1 min and 72 °C for 2.5 min, then terminated by a final extension step at 72 °C for 10 min in an Eppendorf Mastercycler (Eppendorf AG, Hamburg, Germany). The PCR product was subsequently purified with a PCR purification kit and digested with *Nde*I and *BamH*I restriction endonucleases. The restriction fragment was purified by gel extraction kit and then subcloned into the prokaryotic expression vector pET28a to yield pET28a-TmcAHAS, in which the encoded protein was endowed with an amino-terminal His6-tag. The recombinant expression plasmid pET28a-TmcAHAS was then transformed into *E. coli* Rosetta(DE3) competent cells and the transformants were selected on LB plates containing 50 µg/mL kanamycin and 34 µg/mL chloramphenicol. Colonies were selected and screened for recombinant plasmid containing the TmcAHAS encoding gene by colony PCR. The constructed plasmid was further identified by enzyme digesting and DNA sequencing.

### Preparation of the whole cell catalyst overexpressing TmcAHAS and purification of TmcAHAS

LB broth (5 mL × 2) containing 50 µg/mL kanamycin and 34 µg/mL chloramphenicol was inoculated separately with a single transformant obtained above, grown overnight at 37 °C, and subsequently added to LB culture (1.0 L × 2) containing 50 µg/mL kanamycin and 34 µg/mL chloramphenicol, respectively. The culture was incubated at 37 °C, 220 rpm until the OD_600_ reached 0.6 (about 3 h). Then it was further incubated at 25 °C, 200 rpm for 20 min followed by addition of IPTG to a final concentration of 0.5 mM. The incubation was continued at 25 °C, 200 rpm for 4 h before the bacteria were harvested by centrifugation (4 °C, 8,000 rpm, 15 min). The bacteria pellet obtained (wet weight 8 g) was aliquoted into two parts, one of which was stored as a whole cell catalyst in − 80 °C. The other was resuspended in 10 mL of lysis buffer (50 mM NaH_2_PO_4_, 300 mM NaCl, 10 mM imidazole, 5 mM β-mercaptoethanol, pH 8.0) containing 10 mg/mL lysozyme and disintegrated by sonication on ice. The homogenate was then centrifuged for 30 min (10,000 rpm, 4 °C), and the supernatant was loaded onto a Ni–NTA agarose column (1.5 cm × 10 cm polypropylene column with 2 mL of Ni–NTA agarose) that was pre-equilibrated with lysis buffer. The column was first washed with a wash buffer (50 mM NaH_2_PO_4_, 300 mM NaCl, and 60 mM imidazole at pH 8.0, 2 mL × 2), and subsequently eluted with a elution buffer (50 mM NaH_2_PO_4_, 300 mM NaCl, and 250 mM imidazole at pH 8.0, 0.5 mL × 8). The protein samples were analyzed by 12% SDS-PAGE and stained with Coomassie Blue R. The purified protein was dialyzed twice against 50 mM phosphate buffer (pH 8.0, 4 °C, 8 h for each dialysis) or 50 mM Tris–HCl buffer (pH 8.0, 4 °C, 8 h for each dialysis). A final dialysis was performed at 4 °C for 8 h against 50 mM phosphate buffer (pH 8.0) or 50 mM Tris–HCl buffer (pH 8.0) containing 5 mM DTT and 20% glycerol. The protein was aliquoted into 0.5-mL Eppendorf tubes (50 µL each), flash-frozen, and stored at -80 °C. Protein concentrations were determined by the Bradford method using BSA as a standard.

### Western blot analysis

Four to five micrograms of each of prestained protein molecular weight marker and Ni^2+^-His affinity columns purified His_6_-TmcAHAS were respectively subjected to 12% SDS-PAGE and then blotted to a nitrocellulose (NC) membrane. The membrane was blocked with 5% skimmed milk powder in TBST buffer (20 mM Tris–HCl, pH 8.0, 150 mM NaCl, 0.05% (v/v) Tween 20) for 2 h at rt. Subsequent hybridization was carried out with the primary antibody (mouse anti-His Tag monoclonal antibody, 1:2,000 dilution with 5% skimmed milk powder) through a 2 h-shaking incubation at rt followed by washing with TBST buffer (3 × 15 min), then with the second antibody (horseradish peroxidase-conjugated goat anti-mouse IgG, 1: 10,000 dilution with 5% skimmed milk powder) through a 1 h shaking incubation at rt followed by washing with TBST buffer (3 × 15 min). The protein bands on membrane were visualized using Tanon 5200 enhanced chemiluminescence (ECL) detection system (Shanghai, China).

### TmcAHAS activity assays

TmcAHAS activity was measured employing the published procedure with minor modification^37^. The assay mixture (final volume 200 μL) contained 50 mM phosphate buffer (pH 8.0), 5 mM MgCl_2_, 1 mM ThDP, 10 μM FAD, 10 mM pyruvate, and 20 μg of recombinant TmcAHAS. The mixture was incubated at 80 °C for 1 h before it was treated with 200 μL of 0.17% (w/v) creatine and 200 μL of 1.7% α-naphthol (w/v, in 1 M NaOH, freshly prepared) at 60 °C for 15 min and maintained at rt for 15 min. The absorbance of the red solution was measured after 100-fold dilution with ddH_2_O on an Agilent 8,453 UV–vis spectrophotometer at 525 nm. A negative control was run under the same conditions without addition of TmcAHAS protein. The enzyme unit was defined as one μmole of acetolactate produced per min by 1 mg of protein under the optimum conditions.

### Determination of optimum conditions and kinetic analysis

The optimum temperature for TmcAHAS activity was measured at pH 8.0 in 50 mM phosphate buffer within a temperature range from 50 to 100 °C (assay time 1 h). The effects of pH on the enzyme activity were tested at pH values between 6.0 and 9.0 at 80 °C (assay time 1 h), and the following buffers were selected: 50 mM NaH_2_PO_4_ (pH 6.0–8.0) and 50 mM Tris–HCl buffer (pH 7.0 and 9.0). The optimum incubation time was determined in 50 mM phosphate buffer at pH 8.0, 80 °C.

For determining enzyme kinetic parameters, assays containing 1–2 μg TmcAHAS were performed in 50 mM phosphate buffer at optimum temperature and pH for 10 min with the substrate at various concentrations and 10 μM FAD, 1 mM ThDP, and 10 mM Mg^2+^. Initial rates were determined for 2–10% formation of acetolactate. *K*_m_ and *V*_max_ were obtained by fitting the experimental data to the Michaelis–Menten equation using GraphPad Prism. All the measurements were carried out three times and from these values the averages were calculated.

### Stability test of TmcAHAS

Purified TmcAHAS was stored at − 80 °C in either 50 mM phosphate buffer (pH 8.0, containing 5 mM DTT and 20% glycerol) or 50 mM Tris–HCl buffer (pH 8.0, containing 5 mM DTT and 20% glycerol), respectively. Its activity was measured in certain time intervals using the photometric method described above.

### Preparation of AC and 3-hydroxy-2-pentanone (HP)

The syntheses of AC and HP were performed in 5 mL reaction mixture containing 50 mM phosphate buffer (pH 8.0), 5 mM MgCl_2_, 1 mM ThDP, 0.05 mM FAD, 0.5 mM DTT, 100 mM sodium pyruvate (for AC) or 50 mM sodium pyruvate plus 50 mM sodium 2-ketobutyrate (for HP). The reaction was initialized by adding 0.5 mg TmcAHAS or 143 mg whole cell (wet weight) and then the mixture was incubated at 80 °C for 1 h. After cooling down to rt, the reaction mixture was first centrifuged at 7,000 rpm, 4 °C for 10 min to remove the precipitate and then the supernatant was extracted with ethyl acetate (5 mL × 3). The organic layers were pooled and evaporated in vacuum at about 35 °C. The residue was then purified on a silica gel column developed by petrol ether-ethyl acetate. The parts containing the desired product were pooled and concentrated in vacuum. The structure of the product was subsequently elucidated through spectroscopic analyses.

### Preparation of R-PAC

The synthesis of *R*-PAC from pyruvate and BA was performed in 5 mL reaction mixture containing 50 mM phosphate buffer (pH 8.0), 5 mM MgCl_2_, 1 mM ThDP, 0.05 mM FAD, 0.5 mM DTT, 50 mM pyruvate, 5% (v/v) DMSO, and 40 mM benzaldehyde. The reaction was started by adding 143 mg whole cell (wet weight, equivalent to 0.5 mg purified TmcAHAS) and then the mixture was incubated at 80 °C for 1 h. Alternatively, the reaction was initialized by addition of 200 mg whole cell and then the mixture was incubated at 50 °C overnight. Then the reaction mixture was worked up and purified on silica gel column in the same way as for AC and HP, except that the extraction solvent used was chloroform (5 mL × 3).

### Analytical methods

The GC analyses of the reactions for the syntheses of AC and HP were carried out on an Aglient 7820A GC system, equipped with a flame ionization detector (FID) and an Agilent HT-Wax capillary column (30 m × 320 μm × 0.25 μm). Samples for GC measurements were extracted with equal volume of ethyl acetate in advance. The temperatures of the injector and detector were both set at 240 °C. The GC program was set as follows: 40 °C (1 min), ramp to 60 °C (2 min) at 5 °C min^−1^, then a ramp of 10 °C min^−1^ to 160 °C (2.5 min). The HPLC analysis of the reaction for the synthesis of *R*-PAC was performed on an Aglient 1200 HPLC system, equipped with an autosampler and a diode array spectrometer. Separation was carried out on an Agilent RP-18 column (250 × 4.6 mm, 5 μm) with 30% aqueous acetonitrile as an isocratic mobile phase, at a detection wavelength of 280 nm, a flow rate of 0.7 mL/min, and ambient temperature. ^1^H-NMR spectrum was recorded at ambient temperature on a Varian Inova-600 MHz NMR spectrometer. High-resolution mass spectrum (ESI–MS) was obtained using a Thermo Fisher LTQ XL system spectrometer.

Stereoselectivity analyses of the TmcAHAS catalyzed reactions were performed also on the Aglient 7820A GC system equipped with an FID and an Agilent CP-Chirasil capillary column (20 m × 250 μm × 0.25 μm). For AC and HP analyses, the temperatures of the injector and detector were both set at 260°; The GC program was set as follows: 60 °C (3 min), ramp to 80 °C (7 min) at 10 °C min^−1^, then a ramp of 20 °C min^−1^ to 125 °C (10 min)^1^. For *R*-PAC analysis, the temperatures of the injector and detector were both set at 220 °C; The GC program was set as follows: 100 °C (3 min), ramp to 120 °C (30 min) at 5 °C min^−1^. The *R*/*S* ratio of two isomers was calculated from corresponding peak areas^12,38^.

## Supplementary information

Supplementary Information.
