# Supplementary material for: Preparation of a whole cell catalyst overexpressing acetohydroxyacid synthase of Thermotoga maritima and its application in the syntheses of α-hydroxyketones
Source: Sci Rep. 2020 Sep 21;10:15404. doi: 10.1038/s41598-020-72416-6 (PMC7505981; doi:10.1038/s41598-020-72416-6)
Supplement: Supplementary file 1 — Supplementary Information. [file 41598_2020_72416_MOESM1_ESM.doc]

**SUPPORTING INFORMATION**

**Preparation of a whole cell catalyst overexpressing acetohydroxyacid synthase of *Thermotoga maritima* and its application in the syntheses of α-hydroxyketones**

Yan-Fei Liang, Le-Tian Yan, Qiao Yue, Ji-Kui Zhao, Cai-Yun Luo, Feng Gao, Heng Li,* Wen-Yun Gao*

*College of Life Sciences, Northwest University, 229 North Taibai Road, Xi’an, Shaanxi 710069, P. R. China.*

**Construction of expression vector**

The gene (open reading frame) encoding the catalytic subunit of AHAS was amplified from the genomic DNA of *T. maritima* by PCR, and the resulting PCR products were analyzed by 1% (w/v) agarose gel electrophoresis after stained with Goldview. The yielded PCR products were inserted into TA cloning vector pMD18T and then sub-cloned into pET28a, resulting in pET28a-TmcAHAS expression vector that utilizes T7 promoter, potentially allowing high level expression of the desired protein. The recombinant plasmid was further confirmed by *Nde*I and *Bam*HI enzyme digestion and DNA sequencing.


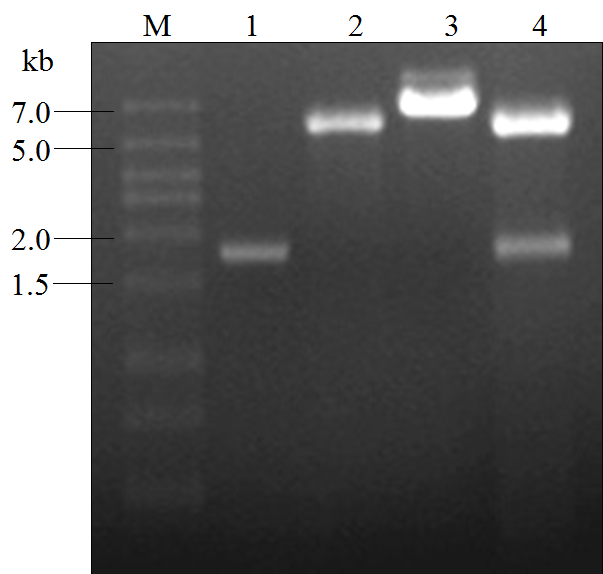


**Fig. S1** The agarose gel electrophoresis result of the preparation of TmcAHAS expression vector. Lane M, DNA maker; lane 1, PCR product of the gene encoding TmcAHAS; lane 2, pET28a vector digested by *Nde*I; lane 3, Recombinant plasmid digested by *Nde*I; lane 4, Recombinant plasmid digested by *Nde*I and *Bam*HI.

**Optimum conditions for TmcAHAS assay**

**Fig. S2** Determination of optimum assay conditions for TmcAHAS. 50 mM buffer containing 5.0 mM MgCl_2_, 1.0 mM ThDP, 10 μM FAD, 50 mM pyruvate, and 10 μg of recombinant TmcAHAS (100 μL). (A) Different buffers (phosphate buffer (PB) or Tris-HCl buffer) at pH 6.0-9.0, 80 ^o^C for 1 h; (B) Phosphate buffer at pH 8.0, 50-100 ^o^C for 1 h; (C) Phosphate buffer at pH 8.0, 80 ^o^C for 0-90 min.


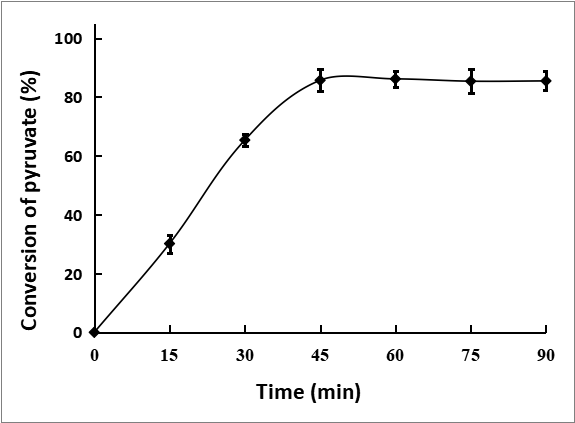

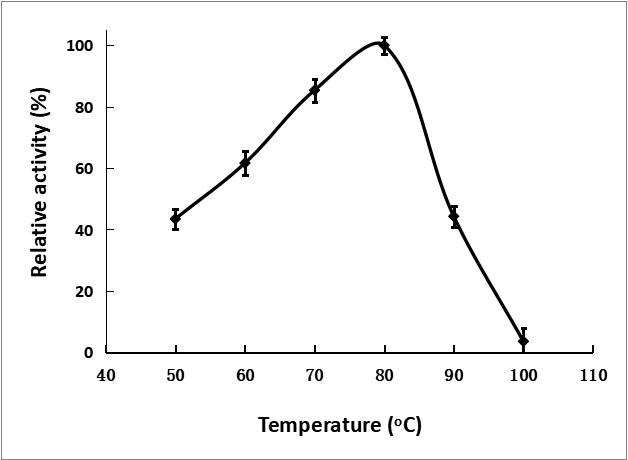

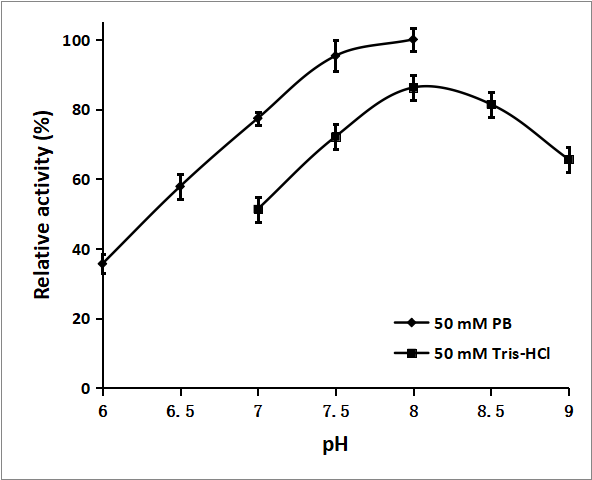


**A**

**B**

**C**

**Determination of the kinetic parameters of TmcAHAS**


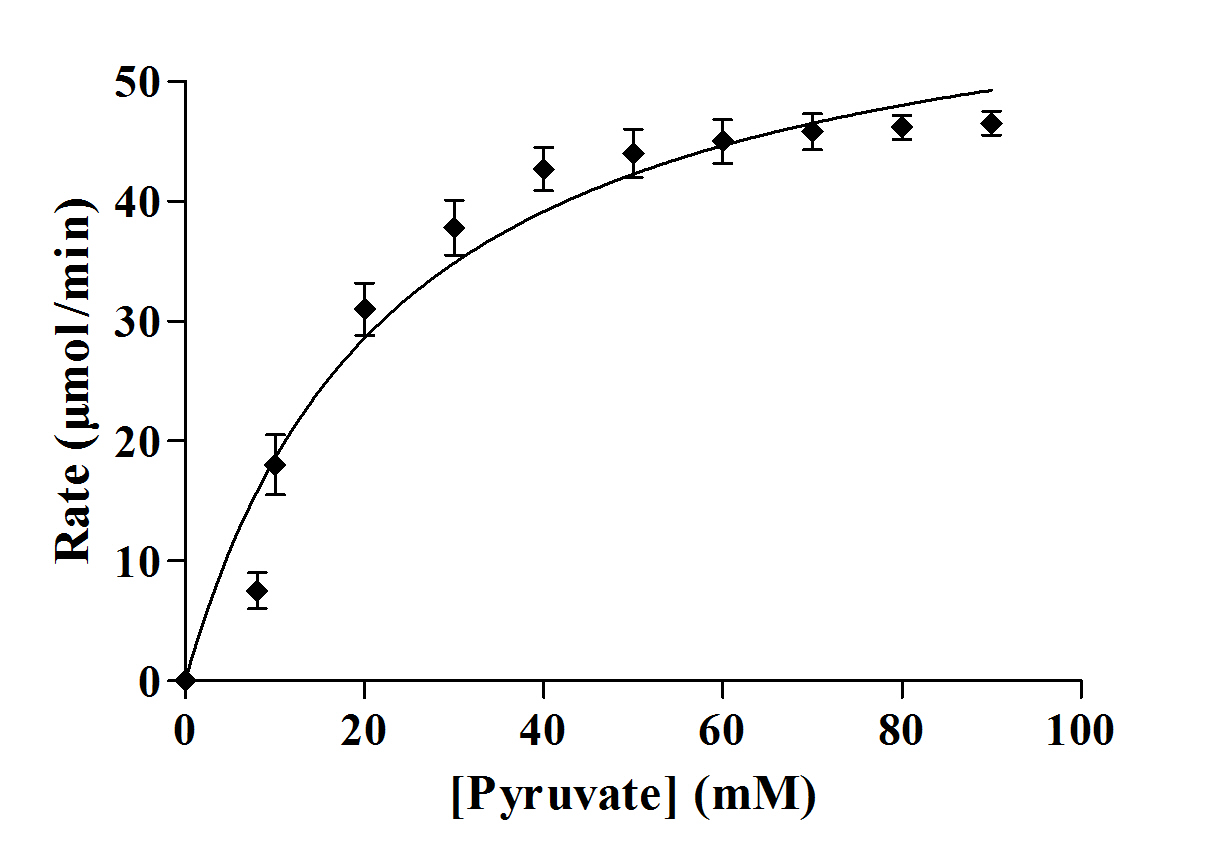


**Fig. S3** Representative Michaelis-Menten kinetic analysis of pyruvate on TmcAHAS. 50 mM phosphate buffer (pH 8.0), 10 μM FAD, 1 mM ThDP, 10 mM Mg^2+^, pyruvate 5-100 mM, TmcAHAS 2 μg, final volume 200μL, 80 ^o^C for 10 min.

**Chiral GC determination of the *R*/*S* ratio of AC**

**Fig. S4** The *R*/*S* ratio of *R/S*-AC determined by chiral GC. The reaction conditions are as follows: 50 mM phosphate buffer (pH 8.0), 5 mM MgCl_2_, 1 mM ThDP, 0.05 mM FAD, 0.5 mM DTT, 100 mM sodium pyruvate, 143 mg whole cell (wet weight) catalyst, 80^o^C for 1 h.

*R*-AC

*S*-AC

**Optimum ratio of pyruvate to 2-ketobutyrate for the preparation of HP**


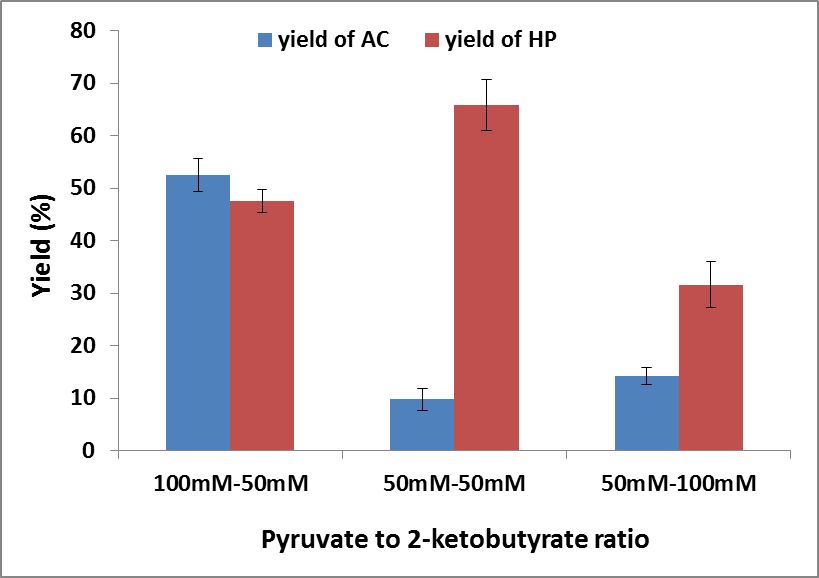


**Fig. S5** Optimization of pyruvate to 2-ketobutyrate ratio for the TmcAHAS catalyzed preparation of 3-hydroxy-2-pentanone (HP). The assay was carried out in 0.5 mL 50 mM phosphate buffer (pH 8.0) containing 0.1 mg TmcAHAS, 80 ^o^C, 1 h. The yields of acetoin (AC) and HP were calculated directly from the GC measurements.

**Chiral GC determination of the *R*/*S* ratio of HP**

**Fig. S6** The *R*/*S* ratio of *R/S*-HP determined by chiral GC. The reaction conditions are as follows: 50 mM phosphate buffer (pH 8.0), 5 mM MgCl_2_, 1 mM ThDP, 0.05 mM FAD, 0.5 mM DTT, 50 mM sodium pyruvate plus 50 mM sodium 2-ketobutyrate, 143 mg whole cell catalyst, 80^o^C, 1 h.

*S*-HP

*R*-HP

**Optimization of reaction conditions for the preparation of *R*-PAC**


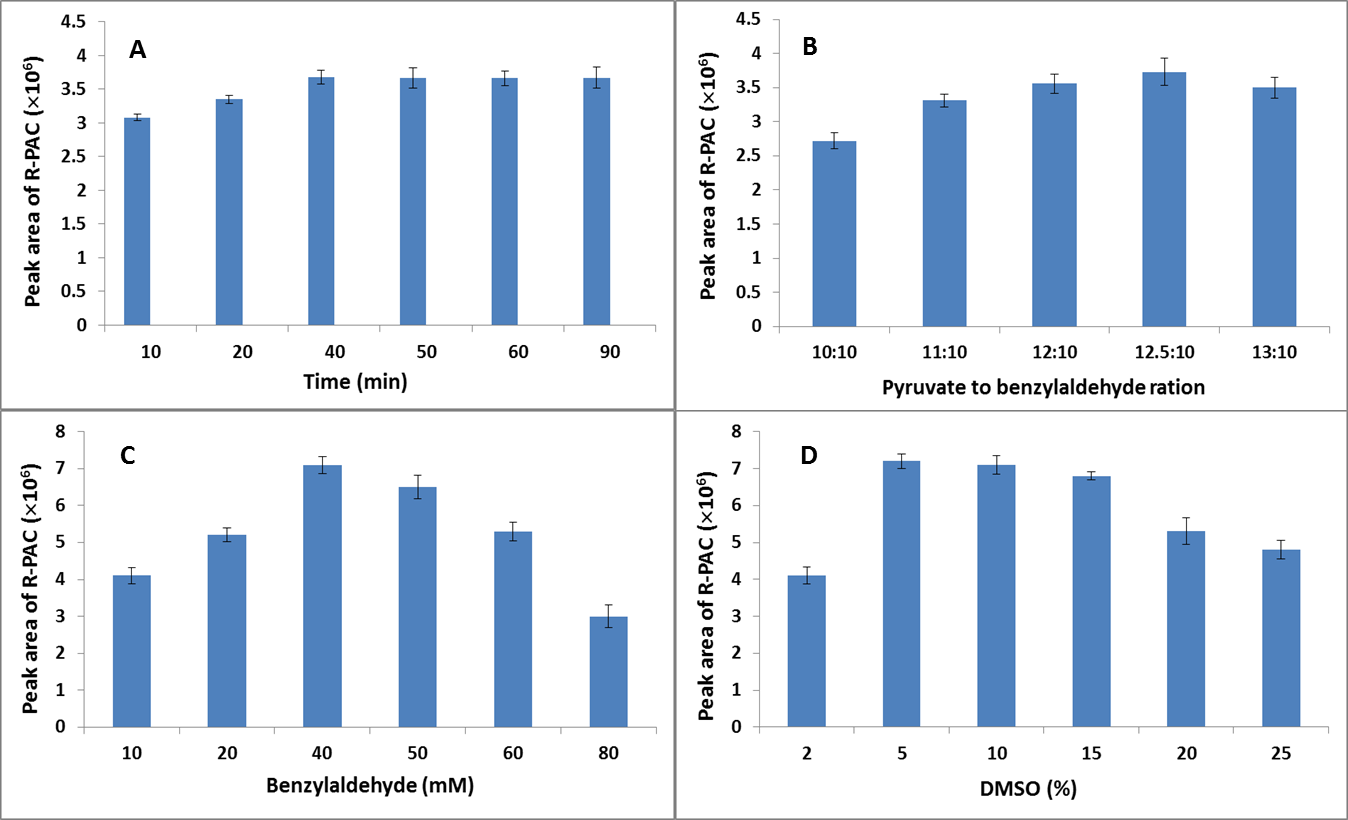


**Fig. S7** Optimization of reaction conditions for the TmcAHAS catalyzed preparation of R-PAC. The peak area determined by HPLC was used to represent the yield of *R*-PAC. The reaction was performed in 0.5 mL 50 mM phosphate buffer (pH 8.0) containing 50 μg TmcAHAS, 80 ^o^C. (A) 12 mM pyruvate, 10 mM benzylaldehyde, 5% DMSO (V/V); (B) 5% DMSO (V/V), 1 h; (C) the concentration of pyruvate was 1.25 times of that of benzylaldehyde, 5% DMSO (V/V), 1 h; (D) 50 mM pyruvate, 40 mM benzylaldehyde, 1 h.

**Comparison of purified protein and the whole cell catalyst in the preparation of *R*-PAC**


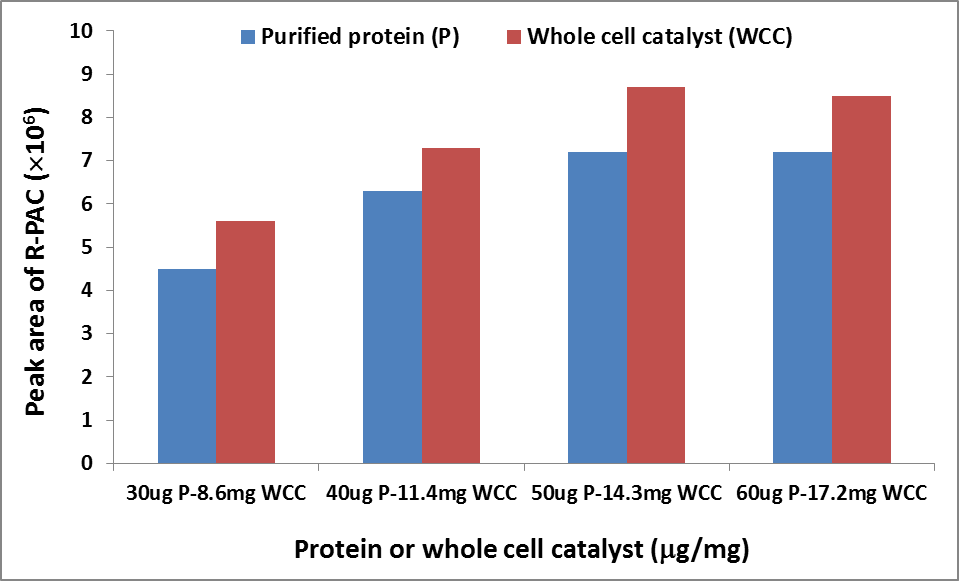


**Fig. S8** Comparison of purified protein and the whole cell catalyst in the preparation of *R*-PAC. The peak area determined by HPLC was used to represent the yield of *R*-PAC. The reaction was performed in 0.5 mL 50 mM phosphate buffer (pH 8.0) containing 50 mM pyruvate, 40 mM benzylaldehyde, 5% DMSO (V/V) and different amount of pure TmcAHAS (P) or the whole cell catalyst (WCC) (2.86 mg WCC is in correspondence to 10 μg pure protein) at 80 ^o^C for 1 h.

**Chiral GC determination of the *R*/*S* ratio of *R*-PAC**

**
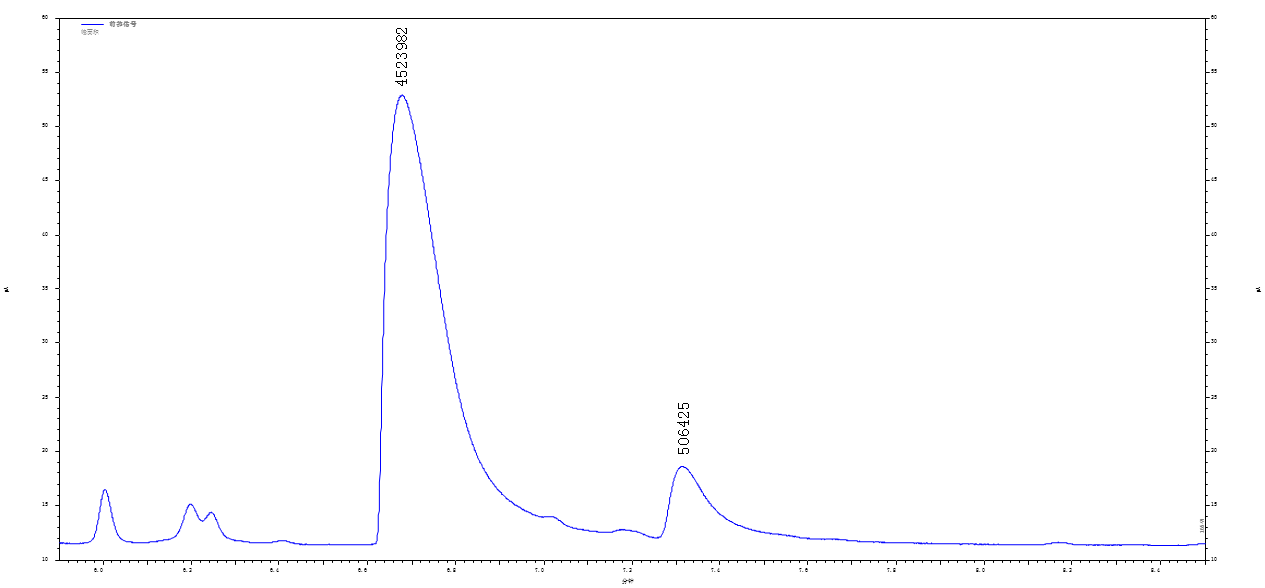
**

*R*-PAC

*S*-PAC

**Fig. S9** The *R*/*S* ratio of *R*-PAC determined by chiral GC. The reaction conditions are as follows: 50 mM phosphate buffer (pH 8.0), 5 mM MgCl_2_, 1 mM ThDP, 0.05 mM FAD, 0.5 mM DTT, 40 mM BA, 200 mg whole cell catalyst, 50^o^C, overnight.
